# African variation at Cytochrome P450 genes: Evolutionary aspects and the implications for the treatment of infectious diseases

**DOI:** 10.1093/emph/eot010

**Published:** 2013-05-15

**Authors:** Ripudaman K. Bains

**Affiliations:** Research Department of Genetics, Evolution and Environment, Darwin Building, University College London, London WC1E 6BT, UK

**Keywords:** Cytochromes P450, sub-Saharan Africa, infectious diseases, evolutionary medicine

## Abstract

The genomics revolution has provided a plethora of data from many previously uncharacterized populations. The increase in the amount of genetic data has improved our understanding of why individuals and populations differ in their susceptibility to multiple diseases. It has also enabled researchers to identify how genomic variation, including at the Cytochrome P450 (CYP450) super-family, affects the safety and efficacy of therapeutic drugs. CYP450 metabolize ∼90% of clinically administered drugs. Variability in CYP450 expression is known to affect the safety and efficacy of therapeutic drugs, including many used in the treatment and control of infectious diseases. There are inter-ethnic differences in the frequencies of clinically relevant CYP450 variants which affect CYP450 expression. Comparative studies of African populations have identified population structuring at CYP450 genes. This is associated with intra-African differences in the success of drug therapies used in the treatment of infectious diseases. Therapeutic drugs dominate control strategies for infectious diseases and are widely administered through mass drug administration campaigns. However, resistance to chemotherapy is spreading across endemic regions. The most common response has been to increase chemotherapeutic dosages, and administer combination therapies. However, there are few pharmacovigilance data examining how these changes influence adverse drug reactions. This review provides an overview of current knowledge of intra-Africa CYP450 variation, and the known associations with sub-optimal clinical outcomes in the treatment of infectious diseases. In addition, the potential for evolutionary approaches in the study of CYP450 variation is discussed to examine their potential in preventative medicine and intervention strategies within Africa.

## OVERVIEW

In recent years, there has been an exponential increase in the amount of genetic data which have made the promise of personalized genomics and translational medicine a reality. Pharmacogenetics studies in particular have identified genetic factors that affect the efficacy and safety of drug treatment. Adverse clinical outcomes, associated with drug therapies, are major contributors to global morbidity and mortality [1]. A key focus of pharmacogenetics research has been to identify clinically relevant biomarkers in genes which encode drug metabolizing enzymes, such as Cytochromes P450 (CYP450) (Box 1). The CYP450 super-family is involved in the metabolism of many therapeutic drugs used to treat a wide spectrum of diseases. Polymorphisms within *CYP450* genes have been attributed to sub-optimal clinical outcomes associated with therapeutic drugs [2] (Table 2). It is hoped that studies of variation at *CYP450* loci will identify biomarkers which can be used to guide individual treatment regimens [3, 4].

**Box 1**

CYP450 are a super-family of haem-containing mono-oxygenases which are found mainly in the liver, although extra-hepatic isoforms exist [5]. CYP450 are involved in the metabolism of multiple endogenous and exogenous compounds [6]. CYP450 mediate oxidation, reduction and hydrolysis reactions which expose or add functional groups to substrates to produce polar molecules [7]. CYP450 are of clinical importance due to their role in the phase I metabolism of over 90% of all clinically administered drugs. The expression of many CYP450 enzymes differs significantly between populations, and their relevance to pharmacovigilance is beginning to be realized.

There are well-known examples of inter-ethnic differences in the frequencies of common and rare genetic markers which influence CYP450 expression phenotypes [8]. However, a number of populations remain under-represented in *CYP450* pharmacogenetics research, including many in sub-Saharan Africa. The importance of including sub-Saharan Africans as study populations within clinical and genomic research should not be underestimated. Approximately 800 million people reside in the sub-continent and are at risk from common and neglected diseases [9, 10]. High levels of genetic diversity are observed within sub-Saharan Africa comparative to other global regions [11], and there are known inter-ethnic differences in the susceptibility to adverse clinical outcomes [12]. However, many sub-Saharan African countries rely on the Food and Drug Administration (FDA) and European guidelines for safety levels and optimal dosages of therapeutic drugs. Therefore, it is important to understand not only how socio-economic factors impact disease burden within the sub-continent but to also identify genetic factors that impact disease progression, transmission and treatment. Increasing numbers of studies are beginning to focus on sub-Saharan African diversity, most recently with the H3 Africa research initiative (http://h3africa.org/). There have been a number of focused studies of *CYP450* variation within sub-Saharan Africa. This review provides an overview of what is known about variation at genes encoding the seven most pharmacologically active CYP450 enzymes within the region. In addition, the clinical implications of *CYP450* variation for the treatment of infectious diseases are assessed, alongside evidence of directional selection at these loci within and outside Africa.

## THE CLINICAL SIGNIFICANCE OF THE CYTOCHROME P450 SUPER-FAMILY

In humans, 57 active CYP450 enzymes have been identified, 7 of which (CYP1A2, CYP2C8, CYP2C9, CYP2C19, CYP2D6, CYP3A4 and CYP3A5) are together involved in the phase I metabolism of more than 90% of clinically administered drugs [13, 14]. More than 300 polymorphisms have been identified in the seven most clinically relevant *CYP450* genes (www.cypalleles.ki.se). There are many different types of polymorphisms that affect CYP450 expression and activity by affecting gene transcription, protein translation and affinity for substrates (Table 1).

**Table 1.** An overview of the types and proportions of variation identified in the most clinically significant CYP450 enzymes

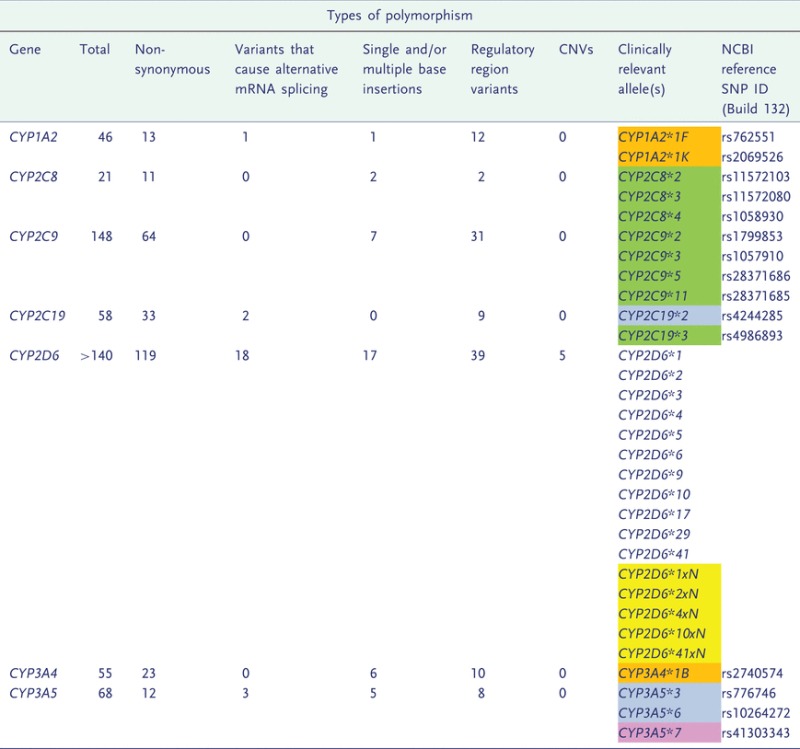

For *CYP2D6*, haplotype information is given, and for all other *CYP450* genes frequencies refer to variant alleles. Numbers in brackets relate to the proportion of each type of variant known to have a functional effect on the CYP450. Information on clinically relevant *CYP450* alleles is also provided, information on their clinical associations is provided in Table 2. Clinically relevant polymorphisms are colour-coded according to whether they are non-synonymous (green), affect mRNA splicing (blue), single/multi-base insertions/deletions (pink), regulatory region variants (orange) or CNVs (yellow).

Not unexpectedly, genetic polymorphisms in *CYP450* genes can affect the efficacy and safety of drug treatment and are associated with adverse clinical outcomes (see Table 2). One important clinical consideration for *CYP450* loci is the considerable substrate overlap between enzymes of this super-family. As a result, the effects of individual CYP450 enzymes on xenobiotic metabolism can be difficult to determine. This is especially true where multiple CYP450 enzymes are involved in the metabolism of a single drug. The effects of polymorphically expressed CYP450 enzymes are easier to elucidate *in vivo*, as it is possible to examine clinical differences between patients who do or do not express a particular enzyme.

**Table 2.** A summary of the most clinically relevant polymorphisms in genes encoding the seven most pharmacologically active CYP450 enzymes, their reported frequencies by geographic region, effects on protein phenotypes and known clinical associations

Enzyme	Contribution to CYP450- mediated metabolism	CYP allele	Effect on enzyme expression	Clinical phenotype	Range of allele frequencies by geographic region^a^	Known clinical associations and effects of allele
CYP1A2	∼9%	CYP1A2*1F	Increased	EM	AFR: 46–66%	Associated with elevated risk of developing tardive dyskinesia in patients treated long-term with anti-psychotic drugs. Recently, it has been shown to increase the metabolism of the anti-psychotic drug olanzapine
					AMR: 73–75%	
					ASN: 59–65%	
					EUR: 62–72%	
		CYP1A2*1K	Reduced	PM*	AFR: 3–15%	Not established
					AMR: 1–5%	
					ASN: 4–10%	
					EUR: 1–6%	
CYP2C8	∼20%	CYP2C8*2	Reduced	PM*	AFR: 15–20%	CYP2C8*2 and CYP2C8*3 are associated with resistance to anti-malarial drugs including chloroquine and amodiaquine. They are also associated with resistance to the anti-cancer drug paclitaxel.
					AMR: 2%	
					ASN: 0%	
					EUR: 1%	
		CYP2C8*3	Reduced	PM*	AFR: 3–5%	
					AMR: 10–17%	
					ASN: 1%	
					EUR: 9–18%	
		CYP2C8*4	Reduced	PM*	AFR: 2%	Similar to CYP2C8*2 and CYP2C8*3 and is also associated with elevated risk of developing Type 2 diabetes
					AMR: 2–4%	
					ASN: 0%	
					EUR: 4–8%	
CYP2C9	∼20%	CYP2C9*2	Reduced	PM*	AFR: 7%	CYP2C9*2 and CYP2C9*3 are significantly associated with elevated risk of excessive anti-coagulation and upper gastrointestinal bleeding risk
					AMR: 10–17%	
					ASN: 1%	
					EUR: 9–16%	
		CYP2C9*3	Reduced	PM*	AFR: 2–3%	
					AMR: 4–8%	
					ASN: 2–6%	
					EUR: 5–11%	
		CYP2C9*5	Reduced	PM*	AFR: 1–3%	CYP2C9*5 and CYP2C9*11 are also associated with excessive risk of excessive anti-coagulation in African-American patients
					AMR: 1%	
					ASN: 0%	
					EUR: 0%	
		CYP2C9*11	Reduced	PM*	AFR: 1–23%	
					AMR: 0%	
					ASN: 0%	
					EUR: 0%	
CYP2C19	∼20%	CYP2C19*2	No detectable levels of enzyme	PM*	AFR: 11–21%	CYP2C19*2 was found to be associated with an elevated risk of severe cutaneous adverse drug reactions in patients treated with the anti-epileptic drug Phenobarbital
					AMR: 13–14%	
					ASN: 32–36%	
					EUR: 7–22%	
		CYP2C19*3	No detectable levels of enzyme	PM*	AFR: 1%	Not established
					AMR: 0%	
					ASN: 5–6%	
					EUR: 0%	
CYP2D6	∼25%	CYP2D6*1	Normal			*CYP2D6* polymorphisms and haplotypes contribute to the variable metabolism of ∼25% of all clinically administered drugs. CYP2D6 is subject to CNVs. Subjects with multiple copies of normal gene copies (i.e. CYP2D6*1xN and CYP2D6*2N) will metabolize drugs more rapidly than normal (UMs), and ideal plasma concentrations of therapeutic drugs will not be achieved at standard dosages. An example is with the pain relieving drug tramadol which, is often prescribed following surgeries. In 174 patients who received the drug, UMs developed respiratory depression following drug administration, due to poor plasma concentrations due to rapid metabolism of the drug.
		CYP2D6*2	Normal			
		CYP2D6*3	No detectable levels of enzyme			
		CYP2D6*4	No detectable levels of enzyme			
		CYP2D6*5	No detectable levels of enzyme			
		CYP2D6*6	No detectable levels of enzyme			
		CYP2D6*9	Decreased			
		CYP2D6*10	Reduced			
		CYP2D6*17	Reduced			
		CYP2D6*29	Reduced			
		CYP2D6*41	Reduced			
		CYP2D6*1xN	Increased			
		CYP2D6*2xN	Increased			
		CYP2D6*4xN	No detectable levels of enzyme			
		CYP2D6*10xN	Reduced			
						In contrast, individuals who lack a functional CYP2D6 protein have been shown to metabolize CYP2D6 substrates at a slower rate (PMs), which increases their chances of adverse drug reactions. An example is the anti-anginal drug perhexiline. PMs of this drug (i.e. those with non-functional copies of the *CYP2D6* gene) have reduced clearance of this drug which, leads to hepatoxicity and peripheral neuropathy.
		CYP2D6*41xN	Reduced			
CYP3A4	∼50%	CYP3A4*1B	Increased	EM	AFR: 66–86%	Associated with the early onset of puberty
					AMR: 6–20%	
					ASN: 0%	
					EUR: 2–4%	
CYP3A5	∼50%	CYP3A5*3	Reduced/Undetectable	PM*	AFR: 4–81%	CYP3A5*3 is associated with elevated risk of salt-sensitive hypertension in individuals with recent African ancestry. It has also been reported to affect the metabolism of the immunosuppressant drug tacrolimus
					AMR: 76–81%	
					ASN: 69–74%	
					EUR: 93–96%	
		CYP3A5*6	Reduced/Undetectable	PM*	AFR: 5–25%	CYP3A5*6 associated with larger tumour sizes in Japanese breast cancer patients
					AMR: 1–5%	
					ASN: 0%	
					EUR: 1%	
		CYP3A5*7	Reduced/Undetectable	PM*	AFR: 0–21%	Not established
					AMR: 0%	
					ASN: 0%	
					EUR: 0%	

Information has been compiled from the literature and from online databases.

^a^Geographic regions are abbreviated as follows: AFR, Africans; AMR, Americas; ASN, Asia; EUR, Europe.

PM* indicates that individuals who are heterozygous for a particular allele have IM phenotypes.

The implementation of genotype guided medicine for individuals is not in widespread clinical use; although physicians are becoming increasingly aware of clinically relevant genetic polymorphisms. A 2006 study by the FDA reported that ∼25% of all prescriptions written in the USA contained pharmacogenetics labelling [15], including for the anti-coagulant warfarin and polymorphisms in the *CYP2C9* gene [16]. Multiple, independent studies have identified an association between decreased expression of CYP2C9, and elevated risk of excessive anti-coagulation and upper-gastrointestinal bleeding [17]. This risk is significantly associated with the CYP2C9*2 and CYP2C9*3 alleles [17]. Both alleles define independent non-synonymous mutations which, impair CYP2C9 activity and lead to reduced substrate binding (CYP2C9*2), or reduced interactions with co-enzymes involved in substrate metabolism (CYP2C9*3) [18]. Patients who carry either, or both, allele(s) require a lower dose to achieve effective anti-coagulation, and reduce the risk of adverse bleeding [19].

### CYP450 clinical phenotypes

Genetic polymorphisms in *CYP450* genes contribute to adverse clinical outcomes due to their effects on enzyme expression and/or activity. Variability in CYP450 expression and function is found to contribute to four clinical phenotypes: poor (PM), intermediate (IM), extensive (EM) and ultra-rapid metabolizers (UMs) [18] (Table 2).

1. PMs have two copies of alleles which reduce/knock out the expression of a particular CYP450. PMs effectively lack a certain enzyme activity and metabolize drugs inefficiently compared with EM, IM and UMs [20].
2. EMs are homozygous for two functional alleles. EMs tend to metabolize a drug rapidly, and often require higher concentrations of an administered drug than IMs and PMs.
3. UMs carry more than two active gene copies. Ultra-rapid metabolism is a result of gene duplication [i.e. copy number variation (CNV) of a gene’s coding and regulatory regions]. The number of gene copies directly correlates with an increase in protein expression levels, and rapid metabolism of substrates.
4. IMs are heterozygous for one copy of a null allele and a functional allele of a certain *CYP450*. This results in a slight decrease in enzyme activity, but typically drug dosages do not need to be adjusted for IMs.

### *CYP450* variation in Africa

The paucity of affordable and efficient testing methods, and the continuous identification of clinically important genetic variants, has delayed the translation of human genetic information into clinical practice and healthcare administration [1, 21]. Population-based studies have been invaluable in filling this gap. Individuals of a given population may have underlying genetic similarities which could potentially distinguish them from other populations. African populations can be structured by cultural, linguistic, phenotypic, ethnic and genetic differences [11]. Within Africa, the borders between nations are often not the best boundaries with which to distinguish different populations, as the partitioning of much of the African continent by colonial powers was relatively recent, and did not account for considerable inter-ethnic diversity within the region [22]. There are known genetic differences between North and sub-Saharan African groups, which are consistent with the Sahara desert acting as a barrier to gene flow across the region [23]. Detailed genetic analyses of populations from East Africa [24], and the Kalahari Desert [25], have also found considerable inter-ethnic diversity within geographic regions, across the continent.

**Figure 1.**
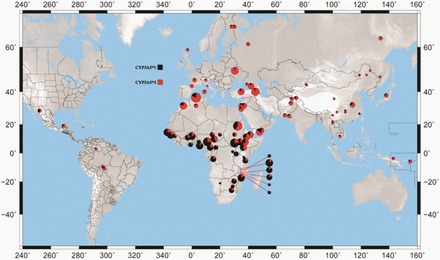
Inferred global CYP3A5*3 allele frequencies using published data. Data for >90 global populations, classified by ethnicity or by language group [29, 30]. The lowest CYP3A5*3 frequencies are found within sub-Saharan Africa, although frequencies are higher in East Africa than in other populations sampled from the continent

The focus on identifying important variants within populations, instead of individuals, has identified differences at *CYP450* loci (see Table 2). One example is the CYP3A5*3 allele, which reduces CYP3A5 expression to undetectable levels [26]. Previous studies have identified differences between individuals with recent African ancestry and European Caucasians, in the frequencies of the CYP3A5*3 allele [27, 28]. A recent study of intra-African variation at the *CYP3A5* gene found considerable differences between populations from the continent [29]. Figure 1 shows CYP3A5*3 allele frequencies for 91 global populations which were previously genotyped [29, 30]. Here, individuals were grouped into populations based on similar languages or by ethnicity. Overall, CYP3A5*3 allele frequencies are lower within Africa than in other global regions. Within Africa, Niger-Congo speaking populations from West, West Central and South East Africa, have very similar allele frequencies. Populations from East Africa are much more heterogeneous. In Ethiopia, CYP3A5*3 frequencies in Afro-Asiatic speaking populations sampled from the north east of the country are comparable to those reported for Yemeni populations. In contrast, CYP3A5*3 frequencies in Nilo-Saharan speakers sampled from the south west of Ethiopia are much more similar to Southern Sudanese groups. North Africans also differ from sub-Saharan African populations [29]. The data shown in Fig. 1 highlight the importance of considering intra-African diversity, as well as inter-continental diversity in *CYP450* genes, given the considerable population structuring within the continent.

### Intra-African *CYP3A* variation

Of all CYP450 enzymes, the CYP3A sub-family (CYP3A4, CYP3A5, CYP3A7 and CYP3A43) play a central role in drug metabolism. CYP3A4 and CYP3A5 are involved in the metabolism of over 50% of all known CYP450 substrates [31], including many used in the treatment of diseases endemic within Africa. There is considerable overlap between CYP3A enzymes in substrate-specificity [32]. Many previous studies have argued that CYP3A4 has the most significant role of all CYP3A enzymes in therapeutic drug metabolism [33]. However, CYP3A5 is one of the most pharmacologically active drug metabolizing enzymes in Africa [29]. Studies have found that CYP3A5 represents at least 50% of the total hepatic and intestinal CYP3A content in individuals who express the protein [32]. This has lead to various studies concluding that variation in the DNA sequence of *CYP3A5* may be the most important genetic contributor to inter-ethnic and inter-population differences in CYP3A dependent drug clearance [34–36].

#### CYP3A5

CYP3A5 is polymorphically expressed between and within ethnic groups. Hepatic and intestinal protein concentrations range from undetectable levels to 202 pmol/mg [26]. Approximately, 10–25% of Europeans, 30–50% of Asian and South Americans and 55–95% of African Americans are predicted to have detectable levels of hepatic and intestinal CYP3A5 protein [26, 27]. Four *CYP3A5* alleles are the most common determinants of inter-ethnic variability in protein expression. The first is CYP3A5*1, the ancestral allele, which defines an expresser phenotype. CYP3A5*3, CYP3A5*6 and CYP3A5*7 each define derived alleles which cause low/non-expresser phenotypes [26, 37–39].

A recent study examined global population differentiation in the frequencies of functionally relevant variants at genes encoding enzymes involved in drug absorption, distribution, metabolism and excretion. The authors found that the most significant inter-population differences were in frequencies of the CYP3A5*3 allele [7]. CYP3A5*3 frequencies differ significantly between ethnic groups, and the mutation is almost at fixation in some European populations [26–28, 37, 38]. The lowest frequencies have been observed in sub-Saharan Africa. However, a recent survey of *CYP3A5* variation within the sub-continent found significant inter-ethnic differences in CYP3A5*3 frequencies across the continent. The authors also identified appreciable frequencies of the CYP3A5*6 and CYP3A5*7 mutations within sub-Saharan Africa. The combined frequencies, and phenotypic effects, of these low/non-expresser mutations suggested that CYP3A5 expression levels across sub-Saharan Africa are likely to be much lower than previously reported, although still higher than in other global regions [29]. The findings of Bains *et al.* suggest that sub-Saharan African patients have an elevated risk of EM phenotypes, compared with populations outside of the sub-continent. Importantly, the findings demonstrate that Africans are likely to be at risk for multiple clinical phenotypes (poor, intermediate and extensive metabolism); when patients are treated with CYP3A drug substrates.

#### CYP3A4

Unlike CYP3A5, CYP3A4 is not polymorphically expressed. Variability in protein expression has been reported, and it is largely attributed to environmental stimuli that affect the regulation and transcription of the gene [40]. The *CYP3A4* gene is characterized by an excess of rare variants, relative to neutral expectations [41], and to date, only one rare mutation has been identified that knocks out CYP3A4 expression [42]. One of the most frequent mutations in this gene is CYP3A4*1B, which occurs in the proximal promoter of *CYP3A4* [43, 44]. The effects of this mutation on enzyme expression are ambiguous. Functional studies have yielded conflicting results, some suggest that the mutation increases enzyme expression [44], although larger studies have not replicated these findings [45]. Therefore, the exact effect of the CYP3A4*1B mutation on CYP3A4 expression, and consequently on clinical phenotypes, is yet to be established.

CYP3A4*1B is often found in high linkage disequilibrium with the CYP3A5*1 allele. Due to the considerable overlap in substrate specificity with CYP3A5 it has been difficult to determine the independent effect of CYP3A4 variability on the safety and efficacy of CYP3A-mediated drug metabolism. It has been reported that for certain drug substrates, the associations between variable CYP3A4 expression and sub-optimal clinical outcomes are not as significant as those seen for CYP3A5 [46]. It is possible that the association between CYP3A variation and clinical phenotypes is a result of variable CYP3A5 expression more than CYP3A4 expression due to the linkage disequilibrium between CYP3A4*1B and CYP3A5*1. This is likely to be the case in populations with recent African ancestry, who are more likely to express CYP3A5 than non-African populations [29]. Further studies will need to examine the extent of linkage disequilibrium between these two mutations across a large African cohort, and how this contributes to the safety of therapeutic drugs in Africa.

#### CYP2D6

CYP2D6 is involved in the metabolism of 25–30% of all CYP450 substrates. CYP2D6 expression is highly polymorphic, and enzyme concentrations contribute to 0–25% of total hepatic CYP450 content [47]. Multiple single nucleotide polymorphisms (SNPs), insertions and deletions (indels), gene conversions and duplications have been identified at this gene. In addition, multiple studies have reported CNV at this locus. Therefore, studies often consider the effects of haplotype compositions on CYP2D6 expression, rather than individual polymorphisms which, often cannot predict expression phenotypes.

Individual haplotypes can increase or decrease CYP2D6 expression (see Table 2). Variability in CYP2D6 expression contributes to multiple clinical phenotypes, including ultra-rapid, poor and extensive metabolism of clinically used drugs [47]. The considerable CNVs that are seen at this locus are of particular interest to clinicians. The most clinically significant are CYP2D6*1xN and CYP2D6*2xN, where *N* refers to the number of copies of a particular haplotype. Both CYP2D6*1 and CYP2D6*2 define normal CYP2D6 expression phenotypes. However, CNVs of these haplotypes can cause UM phenotypes and lead to adverse clinical outcomes [48]. The effects of ultra-rapid metabolism are just as severe as poor metabolic capabilities, only with opposite phenotypes. The number of copies of either a functional or non-functional *CYP2D6* gene significantly influences clinical phenotypes in an additive way (reviewed in [49]).

Considerable variability in CYP2D6 expression phenotypes exists within and between populations [49–51]. Across the 52 populations from the Human Genome Diversity Panel (HGDP), CYP2D6 expression phenotypes were inferred to be largely consistent across non-African groups, although EM phenotypes were not inferred for East Asian populations [52]. North and East Africans were found to have a higher frequency of individuals with increased enzyme activity. Across Africa, there were noticeable regional differences, the proportion of non-expressers was predicted to be higher in West Africa than in other African regions, whereas enzyme expression phenotypes were in the normal range in Southern Africa [51, 52]. However, many indigenous and diverse African populations are not represented on the HGDP, meaning that our understanding of intra-African variation in CYP2D6 expression phenotypes is, at best, incomplete. One recent study found appreciable frequencies (34%) of the CYP2D6*17 allele, which is associated with a significant reduction in enzyme activity, in Zimbabwean populations [53–55]. When these results are considered in the context of those reported for HGDP African groups, they suggest that Southern Africans are likely to have more variability in CYP2D6 expression levels than previously reported. These results also suggest that inter-regional differences are likely to be high across sub-Saharan Africa, and that there is much that still needs to be determined about CYP2D6 variability across the sub-continent.

### The *CYP2C* cluster and the *CYP1A2* gene

The CYP2C sub-family (CYP2C8, CYP2C9, CYP2C18 and CYP2C19) are responsible for the metabolism of ∼20% of all CYP450 substrates [56]. CYP2C8, CYP2C9 and CYP2C19 are the most pharmacologically active enzymes of this sub-family. Each of these three enzymes are polymorphically expressed, and there are inter-ethnic differences in protein expression phenotypes [51, 57, 58]. Interestingly, frequencies of clinically relevant alleles at *CYP2C* genes [58] and inferred expression levels [51] were largely consistent across sub-Africa. However, there are some differences in haplotype structures between populations from the region [56]. The most significant inter-ethnic differences were between sub-Saharan African and non-African populations [56, 57].

Much work has been done to identify common *CYP450* variants, and to identify their global distribution among populations from the HGDP. However, many African populations are under-represented among the 52 groups on the panel. Given the extensive diversity observed within sub-Saharan Africa, many available data and SNP microarrays may not adequately capture diversity at certain loci within the continent. To understand the association between *CYP450* genetic variation and enzyme expression levels within and across Africa, large re-sequencing surveys of these loci are required. One example is the extensive survey of *CYP1A2* variation in diverse Ethiopian populations, which found multiple novel variants in Ethiopia which had not been seen in other global groups, which included the Yoruba from West Africa [59]. Focused re-sequencing surveys will account for population stratification within Africa and are likely to become increasingly important in tailoring drug treatment regimens across the continent. The availability of multiple African data from the 1000 Genomes Project in particular will help to address these issues, and to provide an overview of population stratification at clinically relevant loci in Africa.

### The potential for pharmacogenetics in the treatment of infectious diseases within sub-Saharan Africa

Perhaps the most pressing need for focused pharmacogenetics research in sub-Saharan Africa is to aid the treatment and control of infectious diseases within the region. Drug therapy dominates control strategies for infectious diseases, and many administered drugs are substrates for the seven most pharmacologically active CYP450 enzymes (Table 3). In recent years, there have been widespread mass drug administration (MDA) campaigns to manage disease incidence and prevalence [60, 61]. However, resistance to many drugs used in MDA campaigns is emerging, and established associations between genetic variation and adverse treatment outcomes stresses the need for focused human genetic studies to aid the control of diseases within the region.

**Table 3.** A summary of therapeutic drugs that are used in the control of infectious diseases, and information about CYP450 enzymes that are known to affect their metabolism

Disease	Therapeutic drug	CYP450 enzymes that affect drug metabolism
Malaria	Artemether	CYP2A6, CYP2B6, CYP2C8, CYP2C9, CYP2C19, CYP3A4, CYP3A5,
	Lumefantrine	CYP2D6, CYP3A4, CYP3A5
	Amodiaquine	CYP1A1, CYP1B1, CYP2C8
	Mefloquine	CYP3A4, CYP3A5
	Chloroquine	CYP2C8, CYP2D6, CYP3A4, CYP3A5
	Sulfadoxine-pyrimethamine	CYP2C9, CYP2D6
	Primaquine	CYP1A1, CYP1A2, CYP3A4, CYP3A5,
	Quinine	CYP1A1, CYP1A2, CYP3A5
HIV	Efavirenz	CYP2B6, CYP3A4, CYP3A5
	Saquinavir	CYP3A4, CYP3A5
	Abacavir	No CYP450 enzyme
	Maraviroc	CYP3A4, CYP3A5
	Nevirapine	CYP2B6
	Indinavir	CYP3A4, CYP3A5
	Nelfinavir	CYP3A4, CYP3A5
	Ritonavir	CYP3A4, CYP3A5
	Lopinavir	CYP3A4, CYP3A5
Tuberculosis	Isoniazid	CYP1A2,CYP2C19,CYP2E1
	Rifampin (Rifadin, Rimactane)	CYP1A2, CYP2B6, CYP2C8, CYP2C9, CYP3A4, CYP3A5
	Ethambutol (Myambutol)	CYP2C9, CYP2C19, CYP2E1
	Pyrazinamide	CYP1A2, CYP3A4
Leishmaniasis	Pentostam	No CYP450 enzyme
	Glucantime	No information
	Pentamidine	CYP1A1, CYP1A2, CYP2C8, CYP2C19, CYP2D6, CYP3A4, CYP3A5, CYP4A11
	Amphotericin B	No CYP450 enzyme
	Ketoconazole (on trial)	CYP1A1, CYP1A2, CYP2C8, CYP2C9, CYP2C19, CYP2D6, CYP3A4, CYP3A5, CYP3A7
Human African Trypanosomiasis	Pentamidine (for *Trypanosoma brucei gambiense*)	CYP1A1, CYP1A2, CYP2C8, CYP2C19, CYP2B6, CYP3A4, CYP3A5, CYP4A11,
	Suramin	No CYP450 enzyme
	Melarsoprol	No information
	Eflornithine	No information
	Nifurtimox	No information
South American Trypanosomiasis (Chagas disease)	Benzimidazole	No CYP450 enzyme
	Nifurtimox	No information
Leprosy	Rifampin	CYP1A2, CYP2B6, CYP2C8, CYP2C9, CYP3A4, CYP3A5
	Dapsone	CYP2C8, CYP3A4, CYP3A5
	Clofazimine	CYP3A4, CYP3A5
Lymphatic filariasis	Diethylcarbamazine (DEC)	No CYP450 enzyme

Information has been compiled from the literature and from online databases.

#### Ivermectin and human onchocerciasis

One example of a drug widely used in MDA campaigns is ivermectin, which is used in the treatment of human onchocerciasis (commonly known as river blindness) which is caused by the parasitic worm *Onchocerca voluvulus* and transmitted by blackflies of the genus *Simulium* [62, 63]. Ivermectin is administered multiple times a year for a period of at least 7 years to treat the disease by reducing the parasite load [64], although the drug has varying degrees of success [65]. In recent years, there have been reports of sub-optimal clinical outcomes, specifically the retention of a high parasite load, despite many years of treatment [66]. A recent study of a Ghanaian population found significant associations between reduced treatment efficacy and variants which affect the expression of CYP3A4, CYP3A5, and the drug transporter enzyme P-glycoprotein (encoded by the *MDR-1* gene) [67]. While the sample size was small, this study has highlighted the need to collect data on how genetic factors may influence the safety and efficacy of drugs used in MDA programmes. MDA campaigns do not currently account for inter-population genetic differences at loci which mediate drug metabolism. However, reports of emerging resistance to many chemotherapeutic drugs, widely used in the treatment of infectious diseases across sub-Saharan Africa, suggest that associations between human genetic variation and resistance to drug treatment must be addressed. These studies will highlight the extent of population stratification at these loci across the sub-continent, and identify where drug intervention campaigns may need to be tailored within the region to improve their safety and efficacy.

#### Anti-malaria chemotherapy

In recent years, a number of studies have examined the potential for pharmacogenetics to guide treatment regimens for malaria patients. Malaria remains one of the leading causes of morbidity and mortality within sub-Saharan Africa [68]. Currently, the World Health Organization recommends the use of artemisinin-based therapies as the first-line treatment of *Plasmodium falciparum* infections [69]. Artemisinin was found to have anti-malarial properties in the 1970s and rapidly became a replacement for chloroquine, which is now ineffective against *P. falciparum* malaria [70].

In recent years, there have been increasing numbers of reports of resistance to artemisinin [71, 72]. A number of factors influence resistance to anti-malarial chemotherapy, the most significant is the evolution of drug resistance loci in *Plasmodium* genomes [73, 74]. To help control the spread of drug resistance, artemisinin combination therapies (ACTs) are now administered across malaria-endemic regions [69]. ACT has been very successful in treating *P. falciparum* infections and has contributed to significant decreases in the incidence of severe malaria in endemic regions [75], and in slowing the spread of anti-malarial drug resistance [76]. However, there are reports of resistance to ACT [77]. A number of drugs used for regional control of the disease are CYP450 substrates (Table 3). Little is currently known about the effect of CYP450 variability on the safety and efficacy of ACT. A recent study suggested that CYP450 variability did not significantly affect the efficacy of treatment [78]. However, as resistance to ACT grows across endemic regions, further studies will need to examine how pharmacogenetics factors influence resistance to anti-malarial chemotherapy in addition to parasite resistance.

The most common drugs that are used in combination with artemisinin are amodiaquine, mefloquine and lumefantrine [79]. There are known pharmacogenetics factors that affect the efficacy and safety of these drugs. One of the best studied examples is amodiaquine, which is a substrate for CYP1A1, CYP1A2 and CYP2C8 [80, 81]. Many mild adverse side effects occur as a result of amodiaquine therapy, as well rare severe ones. There is evidence to suggest that adverse reactions are associated with reduced CYP2C8 expression, which causes a PM phenotype [69]. Within Burkina Faso a significant association was observed between frequencies of the CYP2C8*2 variant (which reduces CYP2C8 expression) and the risk of patients developing severe abdominal pain [82]. In addition to mild side effects, there have been reports which suggest that a reduction in CYP2C8 expression causes hepatoxicity and a severe reduction in white blood cell count [83]. Across sub-Saharan Africa, frequencies of the CYP2C8*2 mutation are higher in West Africa than in the East and South East of the continent [84]. This suggests that there may be inter-regional differences in the risk of both mild and severe adverse clinical outcomes associated with amodiaquine treatment. In addition to CYP2C8*2, less frequent variants, such as CYP2C8*3 and CYP2C8*4 have been identified, which both reduce CYP2C8 expression. These variants have been identified at higher frequencies in Zanzibar than in West Africa [85] and are likely to contribute to PM phenotypes within the region. Consistent with data for all *CYP450* loci, this is suggestive of intra-African differences at these genes which may contribute to inter-population differences in clinical outcomes associated with ACT.

#### Additional factors that influence the safety and efficacy of therapeutic drugs

It is important to note that a number of factors contribute to the success of drug therapies. One factor known to affect clinical outcomes in HIV-1 patients is the use of African traditional medicines alongside conventional anti-retroviral treatments [86]. This has highlighted an important area that must also be considered in pharmacovigilance research; understanding how traditional medicine may also affect the efficacy and safety of drug metabolism by interfering with the expression of CYP450 enzymes. There is also the possibility of interactions between administered drugs, whereby one drug interferes with the metabolism of the other. An appreciation of the combined effects of factors which influence drug therapies will help to address the growing problem of drug resistance of multiple infectious pathogens.

### Understanding *CYP450* variation in an evolutionary context

CYP450 are largely studied for their role in drug metabolism; of all human studies of *CYP450* genes (∼40 000), >22 000 are focused studies on particular drug substrates (http://www.ncbi.nlm.nih.gov/pubmed). However, the ability of these enzymes to metabolize drugs is a bi-product of what is believed to be their ‘native’ role. CYP450 paralogues exist in multiple prokaryotic and eukaryotic species and the genes are thought to have existed on the planet for over 2 billion years [87]. It is thought that the ability of CYP450 enzymes to metabolize exogenous compounds evolved 400–500 million years ago to enable animals to digest chemicals in plants, creating water-soluble compounds that are easier to excrete [88]. Human CYP450 enzymes are found in multiple tissues in the body, which include the intestine, lungs and kidneys. However, the majority are found in the liver where they are important in the biosynthesis of bile acids and cholesterol (the most abundant steroid found in animal tissues) [5]. The role of CYP450 enzymes in drug metabolism has arrived very late in human evolutionary history. Despite the considerable substrate overlap between CYP450 substrates, studies have identified evidence of selection on individual, and clusters of, *CYP450* genes, which is discussed below.

### Evidence of selection on *CYP3A* genes

In addition to drug metabolism, CYP3A enzymes are involved in the metabolism of cholesterols, bile acids and steroid hormones [89]. A recent study of the *CYP3A* cluster reported evidence of purifying selection on *CYP3A4* and *CYP3A7* [41]. Low levels of nucleotide diversity and high levels of sequence conservation were observed in the coding regions of these two genes. In contrast, a significant departure from neutrality in the coding regions of *CYP3A5* and *CYP3A43* was observed in Caucasian individuals, consistent with a selective sweep and positive selection. The authors also reported higher frequencies of derived, non-functional *CYP3A5* and *CYP3A43* alleles in Caucasian individuals comparative to Africans.

A previous study reported evidence of positive selection on the *CYP3A* cluster, specifically on the *CYP3A5* gene [30]. CYP3A5 mediates the metabolism of cortisol to 6-β-hydroxycortisol in the kidney, which is important for the retention of water and salt [26, 30]. The authors observed a significant correlation between low/non-expression of CYP3A5 and increased latitude. This pattern is seen for functional variants of genes implicated in increased hypertension risk [90], and the correlation was found to be non-random when compared with neutral markers in the human genome. An independent study also provided strong evidence of a selective sweep/positive selection on the low/non-expresser CYP3A5*3 mutation in populations from the Middle East, Europe and Central South Asia [7], groups which are found at high latitudes.

A recent study examined this association in more detail. Latitude is a correlate of multiple ecological variables which are related to aridity, and Bains *et al.* found that CYP3A5 expression was positively correlated with aridity measures for the present day, Holocene (10 000 years ago) and Late Pleistocene (50 000 years ago) [29]. Theoretically, these results suggest that latitude and aridity measures can be used to predict global CYP3A5 expression phenotypes and identify populations that have an elevated risk of diseases and clinical phenotypes which are associated with differential CYP3A5 expression phenotypes.

### Evidence of selection on *CYP2D6*

There are conflicting reports as to whether the *CYP2D6* gene has undergone positive selection. In 2005, it was suggested that global differences in CYP2D6 expression phenotypes were indicative of selection on the gene in different geographic regions [48]. CYP2D6 is involved in the metabolism of alkaloids, which are found in many foods [91]. It was proposed that the high frequencies of UM in North and East Africa evolved within these geographic regions in response to periods of starvation so that more food would be available [48]. However, in 2006, a large survey of *CYP2D6* variation in 52 populations from the HGDP found that the global distribution of variation, which defined UM phenotypes, did not significantly differ from neutral genomic markers [52]. Additional studies of *CYP2D6* variation also found no evidence of selection on the gene in other African populations, Central [92] and South America [93].

## CONCLUSION

There remains much to be discovered about the extent of global variation at *CYP450* loci. The role of CYP450 in drug metabolism and disease predisposition is well established. However, it is becoming increasingly clear that the enzymes have played an important role in human evolutionary history. Studies have identified correlations between ecological variables and CYP450 expression phenotypes. Theoretically further environmental, evolutionary and demographic considerations of variation at *CYP450* loci will help to map the global distribution of specific adverse clinical outcomes including UM, EM and PM phenotypes. As scientific research becomes increasingly inter-disciplinary, clinically relevant variation will need to be considered in a geographic and evolutionary context and on medical school curriculums. In an increasingly globalized world, considerations of multiple populations and the factors which shape variation and similarities between us will be essential in designing and targeting effective public health programmes. Under-represented populations must be incorporated in genomics studies, not just those of *CYP450* loci, to truly realize the potential for personalized genomics for the world’s most vulnerable populations, many of whom live in regions with a heavy burden of infectious diseases.
